# Exploring the Potential of Chitosan–Phytochemical Composites in Preventing the Contamination of Antibiotic-Resistant Bacteria on Food Surfaces: A Review

**DOI:** 10.3390/molecules30030455

**Published:** 2025-01-21

**Authors:** Nguyen Thi Doan, Nguyen Van Quan, La Hoang Anh, Nguyen Dang Duc, Tran Dang Xuan

**Affiliations:** 1 Graduate School of Innovation and Practice for Smart Society, Hiroshima University, Higashi-Hiroshima 739-8529, Japan; 2 Graduate School of Advanced Science and Engineering, Hiroshima University, Higashi-Hiroshima 739-8529, Japan; 3 Center for the Planetary Health and Innovation Science (PHIS), The IDEC Institute, Hiroshima University, Higashi-Hiroshima 739-8529, Japan; 4 Graduate School of Integrated Sciences for Life, Hiroshima University, Higashi-Hiroshima 739-8529, Japan; 5 Bach Mai Hospital, Hanoi 122000, Vietnam

**Keywords:** chitosan, phytochemicals, antibiotic-resistant bacteria, chitosan–phytochemical composites, food safety

## Abstract

The escalating presence of antibiotic-resistant bacteria (ARB) in food systems presents a pressing challenge, particularly in preventing contamination and ensuring food safety. Traditional sanitation methods, such as cooking and chemical disinfectants, provide effective means to reduce ARB, yet there is a growing need for additional preventive measures directly on food surfaces. This review explores the potential of chitosan–phytochemical composites (CPCs) as surface coatings to prevent the initial contamination of food by ARB, thereby offering a novel complementary approach to conventional food safety practices. Chitosan, combined with active plant-derived metabolites (phytochemicals), forms composites with notable antibacterial and antioxidant properties that enhance its protective effects. We examine CPC synthesis methodologies, including chemical modifications, free radical-induced grafting, and enzyme-mediated techniques, which enhance the stability and activity of CPCs against ARB. Highlighting recent findings on CPCs’ antibacterial efficacy through minimum inhibitory concentrations (MIC) and zones of inhibition, this review underscores its potential to reduce ARB contamination risks on food surfaces, particularly in seafood, meat, and postharvest products. The insights provided here aim to encourage future strategies leveraging CPCs as a preventative surface treatment to mitigate ARB in food production and processing environments.

## 1. Introduction

Recently, issues related to food safety, particularly food poisoning, have become increasingly prominent worldwide. Food poisoning is triggered by the ingestion of contaminated food or beverages containing harmful agents, including bacteria, toxins, metals, and parasites. Among these, bacteria are one of the most common causes of food poisoning. Notable examples include common foodborne pathogens such as *Salmonella* spp., *Staphylococcus aureus*, and *Clostridium perfringens* [1], botulinum-producing strains like *Clostridium botulinum* [2], enteric pathogens [3], and emerging bacterial strains [4]. Antibiotics play a crucial role in preventing and treating bacterial infections, contributing significantly to public health. However, the recent indiscriminate and uncontrolled use of antibiotics has led to the development of antibiotic-resistant bacteria (ARB), affecting foodborne infections. As a result, various pathogens have become resistant to multiple antibiotics, complicating treatment efforts. Typically, *Escherichia coli* has become resistant to several antibiotics, including bactrim, tetracycline, ampicillin/sulbactam, and clindamycin [5]. *Klebsiella pneumoniae* exhibits high resistance to penicillin, cephalosporin, carbapenem, and fluoroquinolone [6], while *Salmonella* is resistant to fluoroquinolone [7]. Moreover, the so-called “ESKAPE” pathogens, which include *Enterococcus faecium*, *S. aureus*, *K. pneumoniae*, *Acinetobacter baumannii*, *Pseudomonas aeruginosa*, and *Enterobacter* spp., are experiencing growing multidrug resistance and virulence [8]. This growing resistance makes it imperative to find new ways to prevent the growth of pathogenic bacteria, including antibiotic-resistant strains, to ensure food safety and alleviate the burden on the health and economic sectors.

Traditional sanitation methods, such as cooking and the use of chemical disinfectants, are foundational in food preservation, helping to prevent spoilage and extend shelf life. Cooking utilizes high temperatures to destroy bacteria, viruses, and fungi, which effectively reduces the risk of contamination and enhances food safety [9]. Chemical disinfectants, including chlorine [10], hydrogen peroxide [11], and other antimicrobial agents, are commonly applied in food processing environments to sanitize surfaces, equipment, and fresh products. These methods are widely employed due to their proven effectiveness in preventing microbial contamination [9,10,11]. However, they also present several limitations. Cooking can alter the texture, flavor, and nutritional value of food, which may reduce the overall quality of the product [9]. In addition, cooking may not completely destroy bacterial toxins, such as enterotoxins, produced by bacteria growing in food, as enterotoxins remain stable at 100 °C for over an hour [12]. Chemical disinfectants may leave residues that pose potential health risks to consumers or contribute to environmental pollution [11]. Therefore, natural and safe preservatives that enhance food safety without compromising quality are needed.

The preservation of food is an ongoing battle against microbial agents that threaten its safety. The food industry is increasingly exploring alternatives to traditional preservation techniques, driven by consumer demand for safe, natural, and convenient food products. Among these emerging strategies, the use of natural preservatives stands out as the most extensively investigated approach. Studies have shown the efficacy of natural compounds from plants for antimicrobial purposes, including against ARB. For instance, it is shown that sterols and hexadecenoic acid, present in *Tridax procumbens* L., exhibit effectiveness against bacterial strains such as *E. coli*, *S. aureus*, *Bacillus subtilis*, and *Proteus mirabilis* [13]. Other examples include methyl gallate and fraxetin, found in the stem bark of *Jatropha podagrica*, along with cordycepin in *Cordyceps militaris*, which have demonstrated efficacy against both *E. coli* and *B. subtilis* [14,15], and terpenoids from *Perilla frutescens* leaves, which exhibited effectiveness against a broad spectrum of microbes [16]. Additionally, momilactones, a diterpenoid lactone abundant in rice by-products like straw and husks, has been found to significantly inhibit the growth of *E. coli*, *P. ovalis*, *B. cereus*, and *B. pumilus*, as well as certain harmful fungi [17]. These findings highlight the potential of natural preservatives in enhancing food safety, particularly against antibiotic-resistant pathogens.

On another note, chitosan, a deacetylated derivative of chitin obtained from shrimp and crab by-products, is widely recognized for its inherent antimicrobial properties [18,19,20]. Numerous studies have explored grafting natural compounds onto chitosan to enhance its effectiveness against ARB. For example, gallic acid grafted chitosan inhibits *S. aureus*, *B. subtilis*, *B. cereus*, *E. faecalis* [21], caffeic acid grafted chitosan inhibits *Propionibacterium acnes*, *Staphylococcus epidermidis*, *S. aureus*, *P. aeruginosa* [22], and quercetin-modified chitosan mitigates *E. coli*, *S. aureus*, and *P. aeruginosa* [23].

The application of CPCs in the context of food safety embodies dual functionality. Primarily, these conjugates are harnessed as natural preservatives in the domain of food processing, manifesting inhibitory efficacy against both pathogenic and antibiotic-resistant bacterial strains. This phenomenon significantly extends the shelf life of food products by enhancing their quality properties, thereby contributing to food safety. Secondarily, the conjugates assume a pivotal role as integral components of food packaging materials, imparting an enhanced stratum of protection against bacterial contamination throughout the stages of storage and transportation. Due to the unique structure of a linear polymer, chitosan can form a stable structural system with phytochemicals through covalent and non-covalent bonding, such as Schiff base formation (-C=N-), hydrogen bonding, and ionic interactions. These interactions result in a stable composite system that enhances the bioavailability and bioactivity of phytochemicals while improving the antimicrobial efficacy of CPCs. In addition, the surface behavior and stability of CPCs are critical for their application as food coatings or films, particularly in maintaining food quality during storage. Specifically, with properties such as film-forming, thermal stability, moisture resistance, adhesion and surface uniformity, and high surface compatibility with food matrices, CPCs are expected to be a highly versatile material that extends shelf life, reduces spoilage, and enhances the safety of fresh and minimally processed foods.

With the growing threat of ARB and the pressing need for innovative food preservation methods, this paper aims to explore the potential of CPCs as a solution to address both challenges. By reviewing the current research, we provide insights into the development of CPCs as surface coatings to protect food from ARB, offering a viable alternative to conventional preservation technologies. This approach not only addresses critical public health concerns but also aligns with industry trends toward natural antimicrobial solutions that enhance food safety and reduce the risk of antimicrobial resistance.

## 2. Materials and Methods

In this study, we gathered documents retrieved from reputable databases, specifically Web of Science, ScienceDirect, Scopus, and the National Library of Medicine (via PubMed), spanning the timeframe from 2000 to 2024. The searching strategy focused on the keywords “antibiotic-resistant bacteria”, “chitosan–phytochemical composites”, and “food safety”, aiming to identify documents pertaining to the utilization of CPCs as a potential strategy against ARB associated with foodborne illnesses. The investigation delved into various techniques for synthesizing chitosan–phytochemical conjugates, including chemical modification, grafting, and enzyme-mediated techniques. Furthermore, it assessed their inhibitory effects on antibiotic-resistant bacterial strains and explored the applications of CPCs in food preservation. A total of 250 scientific publications were screened and filtered, resulting in 176 articles being included in the final review. Of these, 53 articles were experimental studies focused on CPCs targeting ARB. The remaining 123 articles contributed to the broader context of the paper, including the introduction and other sections not directly related to experimental studies.

## 3. Antibiotic-Resistant Bacteria in Food: A Global Public Health Crisis

Food contamination with ARB and antibiotic residues is a global concern [24]. First, even in countries with advanced food safety systems and strict regulations, contamination can still occur. This includes the presence of harmful bacteria, including ARB, that can survive throughout the food production and distribution chain. Factors such as improper handling, cross-contamination during processing, and breaches in hygiene protocols can contribute to such incidents. Additionally, the global nature of food trade allows contaminated products to spread across borders, posing significant public health risks [25]. Second, antibiotic residues themselves present a serious food safety issue. Beyond promoting resistance, they also pose allergenic risks. Consuming food containing antibiotic residues can expose people to subtherapeutic doses of antibiotics, facilitating the development of resistant bacteria in humans. Moreover, some individuals may be allergic to specific antibiotics, and even trace amounts of residues in food can trigger allergic reactions, ranging from mild symptoms to severe anaphylaxis [26].

Antibiotic resistance poses a widespread global public health crisis characterized by the resilience of bacteria, fungi, and other microorganisms against the effectiveness of antibiotics [27]. This phenomenon amplifies the complexity of infection treatment, resulting in prolonged illnesses, heightened healthcare costs, and, potentially, fatalities [28]. Antibiotic resistance primarily stems from the imprudent overuse and misuse of antibiotics, fostering the evolution of certain bacteria and the emergence of resistance mechanisms [29]. These mechanisms manifest through alterations in the bacterial genome, the acquisition of resistance genes via horizontal gene transfer, or the activation of pre-existing resistance genes [30,31,32].

The multifaceted nature of antibiotic resistance represents a significant and multidimensional threat to public health. Firstly, elevated morbidity and mortality rates arise from the heightened complexity associated with treating resistant infections [33]. Secondly, patients with antibiotic-resistant infections often require extended hospitalization periods, leading to increased healthcare costs. Furthermore, the management of antibiotic-resistant infections results in greater financial expenditure due to the use of alternative, frequently more expensive, antibiotics [34]. The propagation of resistance exacerbates the scarcity of effective antibiotics for infection treatment, potentially leading to the emergence of untreatable conditions in the absence of accessible and efficacious antibiotics [35]. Moreover, antibiotic resistance introduces complications to surgical procedures, cancer treatments, and other medical interventions reliant on robust infection control measures. Within the context of global health threats, the facile dissemination of resistant bacteria across borders presents a formidable international health concern. International travel and trade expedite the rapid global spread of antibiotic-resistant strains [27,36].

Antibiotic use in food production: The utilization of antibiotics in food production is a noteworthy contributor to the escalating concern surrounding antibiotic resistance—a critical global public health issue. Antibiotics find extensive application in agriculture and animal husbandry for various purposes, primarily to prevent and treat bacterial infections in farm animals [37]. In settings characterized by high population density and insufficient sanitation, common in numerous industrial-scale farming operations, antibiotics play a pivotal role in mitigating the transmission of diseases among animals [38]. Additionally, the incorporation of subtherapeutic doses of antibiotics into animal feed for growth promotion, particularly in poultry and livestock farming, has become pervasive [36,38].

Consequences of routine antibiotic applications in food production: The routine application of antibiotics in food production yields several consequences that contribute to antibiotic resistance. Table 1 demonstrates that pathogenic bacteria found in food exhibit resistance to multiple commonly used antibiotics.

The consistent use of antibiotics in animal agriculture exposes bacteria in these environments to protracted periods of low antibiotic concentrations, creating selective pressure that favors the survival and proliferation of ARB. Subsequently, these resistant bacteria may infiltrate the food chain, potentially reaching consumers and facilitating the dissemination of resistance [37,38]. The second factor underscores the presence of resistant bacteria in food items, including meat and dairy products, which act as reservoirs of infection for humans. Consumption of contaminated food places individuals at risk of acquiring infections from these strains, thereby reducing the efficacy of antibiotic therapies [48]. Amidst the escalating challenge of antibiotic resistance, chitosan and its derivatives have emerged as a promising alternative in the ongoing battle against ARB.

## 4. Chitosan and Its Antibacterial Characteristics

### 4.1. Introduction of Chitosan

**Figure 1:**
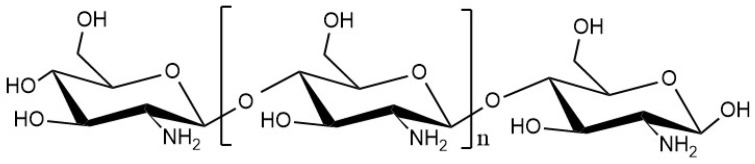
Chitosan’s chemical structure.

Chitosan is a biopolymer derived from chitin, a naturally occurring polymer found in the shells of crustaceans such as shrimp, crab, and lobster, as well as in the cell walls of fungi, insects, algae, and microorganisms [49]. It is produced through the deacetylation of chitin, a process that removes acetyl groups from the chitin molecule, resulting in the formation of chitosan. Chitosan comprises glucosamine units joined by β-(1,4)-glycosidic bonds (Figure 1).

Chitosan is characterized by its functional groups, such as amine, hydroxyl, and acetylated amine groups. These functional groups confer a variety of biological properties, including film-forming capabilities, hypolipidemic activity, biodegradability, antimicrobial activity, immunoadjuvant activity, and acceleration of wound healing [50,51,52]. Notably, the amine functional group (-NH_2_) has garnered significant attention from the scientific community, as it enhances solubility in acidic environments, facilitates chemical modifications by reacting with various compounds, and contributes to antibacterial activity by interacting with bacterial cell membranes [53]. The inherent attributes of chitosan directly relate to the scope of its applicability, as illustrated in Table 2.

Due to its inherent nature as a biopolymer with substantial molecular weight, chitosan, when dissolved in a solvent, produces a high-viscosity gel-like solution. This solution subsequently facilitates the development of a thin film upon deposition or spraying onto material surfaces. The resultant chitosan film exhibits durability, resistance to tearing, and a capacity for biodegradation [62]. Chitosan degradation primarily occurs through enzymatic processes, with lysozyme and chitinase playing pivotal roles. It is noteworthy that lysozyme is abundantly present in the human body, particularly within the pulmonary system, making it a viable material for both drug delivery and medical applications [57]. The positively charged amino groups of chitosan can establish bonds with negatively charged molecules, including lipids and bile acids. Consequently, these complexes may be excreted in the feces. The interaction between chitosan and oil droplets influences the digestibility of the oil. The presence of chitosan reduces the accessibility of oil to digestive enzymes, resulting in a decrease in oil digestion [63]. Additionally, chitosan features numerous -NH_2_ groups, making it soluble in acidic solutions and imparting positive chargeability. The antimicrobial attributes of chitosan can be explained by the interaction between the positively charged ions on chitosan molecules and the negatively charged entities on the membranes of microorganisms.

### 4.2. Antibacterial Mechanisms of Chitosan

Chitosan is well-known for its antibacterial properties [19,64], but the action mechanisms differ from conventional antibiotics [30]. Resistance against antibiotics occurs because these substances have specific molecular targets within bacterial cells [65]. Bacteria can acquire mutations in these specific targets or develop mechanisms to bypass them, leading to resistance [66]. On the other hand, chitosan has been reported as a multi-target compound, interacting with different cellular processes simultaneously. This may make it challenging for bacteria to develop resistance, as it would require simultaneous mutations in various pathways [65,67]. To date, there has been no evidence that chitosan specifically can cause or contribute to antibiotic resistance in bacteria. Further research is needed to better understand this issue. In this section, we discuss the action mechanisms by which chitosan inhibits bacteria to highlight its great potential in addressing the increasingly serious issue of ARB.

**Figure 2:**
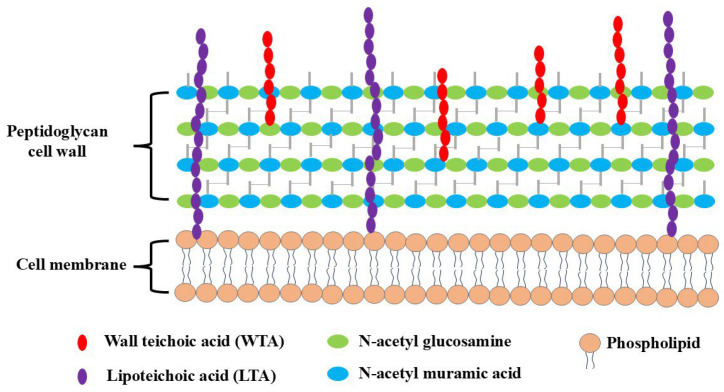
Teichoic acid polymers are located within Gram-positive cell wall.

Chitosan and its derivatives exhibit distinct mechanisms of action against Gram-positive and Gram-negative bacteria [18,19]. This divergence in mechanisms can be attributed to variations in the composition of the bacterial cell wall. In Gram-positive bacteria, the cell wall consists of peptidoglycan, wall teichoic acid (WTA) covalently linked to peptidoglycan, and lipoteichoic acid (LTA) anchored to the microorganism’s cell membrane. Both WTA and LTA feature a negatively charged anionic structure. These teichoic acids create a densely arranged layer of negative charges in the cell wall, which, in turn, inhibits the passage of ions across the membrane, as shown in Figure 2.

**Figure 3:**
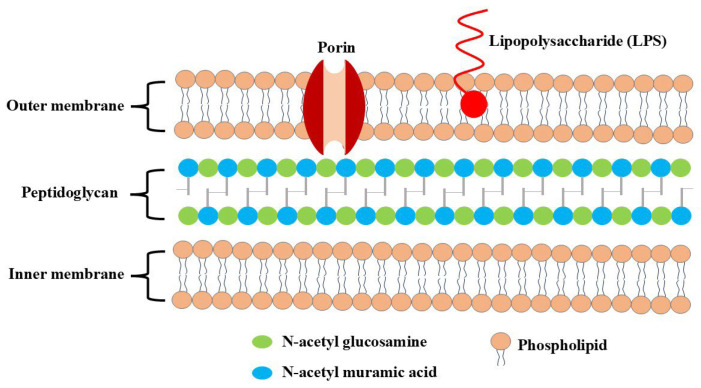
Cell envelope of Gram-negative bacteria.

In Gram-negative bacteria, the cell envelope is composed of two membranes separated by a periplasmic space that includes a thin layer of peptidoglycan. As depicted in Figure 3, the lipid makeup of the outer membrane in Gram-negative bacteria displays an imbalance: lipopolysaccharide (LPS) is predominantly located in the outer layer, while the inner layer is composed of a diverse array of phospholipids. The outer layer of Gram-negative bacteria is characterized by negative charges stemming from the phosphate and pyrophosphate groups of LPS present in the outer membrane. Chitosan’s antimicrobial effects are commonly explained through four widely accepted models.

#### 4.2.1. Membrane Disruption

Chitosan is known to disrupt the integrity of microbial cell membranes. The positively charged amino groups in chitosan interact with the negatively charged components of microbial cell membranes, such as LPS in Gram-negative bacteria or teichoic acids in Gram-positive bacteria. This interaction can lead to membrane permeabilization, compromising the structural integrity of the microorganism and resulting in cell death [18,68].

#### 4.2.2. Interaction with Microbial DNA

Low-molecular-weight (LMW) chitosan and its hydrolyzed derivatives can enter cell walls and impact DNA/RNA as well as protein synthesis [69]. The binding of oleoyl–chitosan nanoparticles (OCNPs) to DNA/RNA was investigated by assessing their impact on the electrophoretic mobility of nucleic acids [70]. The results demonstrated that as the concentration of OCNPs increased, interactions among bacterial genomes intensified. When the OCNP concentration reached 1000 mg/L, the migration of *E. coli* and *S. aureus* DNA and RNA was completely inhibited. This inhibition is presumed to arise from the interaction between the negatively charged phosphate groups in nucleic acid chains and the positively charged amino groups in OCNPs, thereby affecting the pathogen [70]. In another study, the effect of chitosan on protein biosynthesis was examined by inhibiting β-galactosidase expression [71].

#### 4.2.3. Formation of a Polymer Film on the Surface of Microorganisms

Chitosan, with a high molecular weight, can form a dense polymer film on the cell surface, covering the porins on the outer membrane of Gram-negative bacteria. This process obstructs the exchange of nutrients and the uptake of oxygen, ultimately leading to the death of microbial cells [72]. The presence of this film is evident through the visibly thicker cell walls, indicating the deposition of chitosan on the cell surface [73].

#### 4.2.4. Chelation of Nutrients by Chitosan

Both Gram-positive and Gram-negative bacterial cell wall components have an affinity for divalent metal cations essential for microbial cell vitality because they maintain enzymatic functions and the stability of cytoplasmic membranes. WTAs in the peptidoglycan layer of Gram-positive bacteria, particularly attracting Mg^2+^ and Ca^2+^ cations, are crucial for maintaining enzymatic functions and membrane integrity. LPS in Gram-negative bacterial cell surfaces, contributing to the negative charge of the bacterial cell membrane, also exhibits a strong affinity for divalent cations [64,69,72]. The absence of these cations renders bacteria more susceptible to chemicals or certain antibacterial agents. At a pH lower than 6.0, the protonated amino groups in the chitosan polymer chain compete with divalent cations for the phosphate groups present in LPS or teichoic acid structures. The addition of Mg^2+^ and Ca^2+^ to the culture medium increases the membrane’s positive charge, thereby diminishing the antimicrobial efficacy of chitosan [55]. However, when the pH value of the medium is higher than the pKa of chitosan, the amino groups of chitosan become unprotonated amino groups. Therefore, they can donate their lone pair of electrons to the metal ions on the cell surface [74,75].

## 5. Phytochemicals: An Overview

This review emphasizes the use of phytochemicals over synthetic antibiotics or chemicals in developing chitosan composites to address contamination by ARB on food surfaces. The preference for phytochemicals is based on several critical factors. Synthetic antibiotics often impose selective pressure on microbial populations, promoting the emergence and proliferation of ARB, thereby exacerbating the antibiotic resistance crisis [76]. Moreover, synthetic chemicals are associated with potential health risks, including toxicity, allergenicity, and carcinogenic properties. In contrast, phytochemicals derived from plants are generally considered safer alternatives with fewer side effects [77]. In addition to antimicrobial effects, phytochemicals offer dual benefits. They not only exhibit antimicrobial properties but also possess antioxidant activity, which helps prevent food spoilage by inhibiting oxidative processes. These advantages are rarely observed with synthetic antimicrobials [78].

Phytochemicals are naturally occurring metabolites found in plants, commonly referred to as bioactive compounds [79]. These compounds contribute significantly to the diverse array of flavors, colors, and aromas present in fruits, vegetables, grains, legumes, nuts, and other plant-based foods. In addition, phytochemicals encompass a broad spectrum of compounds that help plants to defend against environmental stresses and to attract pollinators [80]. Moreover, phytochemicals play a crucial role in promoting human health and are gaining attention for their potential applications in the food industry, particularly in antimicrobial activities.

Phytochemicals are classified into several categories based on their chemical structures and functional properties. Among known phytochemicals, phenolic compounds (e.g., flavonoids, phenolic acids), alkaloids (e.g., caffeine, nicotine), terpenoids (e.g., carotenoids, terpenes), and organosulfur compounds are the most common compounds identified in plant sources [79]. These natural compounds exhibit a myriad of bioactivities that contribute to human health-promoting effects. Phytochemicals, similar to chitosan, are multi-target compounds with the ability to inhibit bacteria by simultaneously affecting different pathways, which challenges bacteria to develop various mutations to resist [65]. There have also been no reports of ARB against phytochemicals. Furthermore, the combination of chitosan and phytochemicals may offer a synergistic inhibitory effect on bacteria by increasing action targets, making it more effective in overcoming antibiotic resistance [81]. This section highlights phytochemicals with potential antibacterial properties that can be combined with chitosan to achieve synergistic effects against ARB. Table 3 provides an overview of commonly studied phytochemicals and their antibacterial activities.

Numerous phytochemicals, as shown in Table 3, exhibit antioxidant properties, aiding in the neutralization of detrimental free radicals within the body and diminishing oxidative stress. This antioxidant activity is pivotal in mitigating the risk of chronic ailments like cancer, cardiovascular diseases, and neurodegenerative disorders [95]. Consumption of phytochemical-rich foods has been associated with numerous health benefits. For example, previous studies have shown that diets high in fruits, vegetables, whole grains, and legumes, which are abundant sources of phytochemicals, are linked to a reduced risk of chronic diseases such as heart disease, stroke, diabetes, and certain types of cancer [96].

In the food industry, phytochemicals serve various purposes, including enhancing the nutritional value, flavor, color, and shelf-life of food products [97]. Additionally, their antimicrobial properties make them valuable natural preservatives for extending the storage stability of perishable foods and inhibiting the growth of foodborne pathogens and spoilage microorganisms. For instance, essential oils containing phytochemicals such as thymol, carvacrol, and eugenol have been shown to exhibit strong antimicrobial activity against a wide range of bacteria, fungi, and viruses [98]. These natural compounds can be incorporated into food packaging materials and coatings or directly added to food formulations to inhibit microbial growth and prolong the freshness of food products without the need for synthetic preservatives. Moreover, plant-derived extracts rich in polyphenols, such as green tea extract and grape seed extract, have been used to prevent lipid oxidation and microbial spoilage in meat, poultry, and seafood products. Their antioxidant and antimicrobial properties not only maintain the quality and safety of food but also meet consumer demand for clean-label and minimally processed foods [99].

Several studies have elucidated the primary mechanisms by which phytochemicals inhibit ARB. These mechanisms include membrane permeability alteration, efflux pump inhibition, enzyme inhibition, and plasmid curing [100]. Some ARB strains tend to form non-permeable membranes or modify their cell wall structures to reduce the permeability of antibiotics. Research has shown that certain phytocompounds, such as flavonoids, terpenoids, and hydrophobic compounds (e.g., essential oils), can interact with membrane proteins or lipids, leading to structural changes in the membrane and, ultimately, creating a permeable membrane for antibiotics [101]. Efflux pump inhibition is another critical mechanism by which phytochemicals combat ARB. Bazzaz et al. demonstrated decreased efflux of ethidium bromide in the presence of galbanic acid (a sesquiterpene coumarin from *Ferula szowitsiana* roots) in six drug-resistant strains of *S. aureus* [102]. Inhibition of efflux pumps in ARB strains prevents the expulsion of antibiotics, allowing them to remain inside the bacterial cells longer. Moreover, some drug-resistant strains carry antibiotic-modifying enzymes that can degrade or metabolize antibiotics. Allicin (diallyl thiosulfinate), found in *Allium sativum*, has been reported to enhance the activity of ciprofloxacin, tobramycin, and cefoperazone against *P. aeruginosa* by inhibiting sulfhydryl-dependent enzymes such as RNA polymerase, thioredoxin reductase, and alcohol dehydrogenase [103]. Of particular interest is the fact that the primary reservoirs of genes encoding antibiotic resistance in ARB strains are plasmids. Plant-derived chemicals and extracts have shown significant efficacy in curing ARB plasmids. For example, 8-epidiosbulbin E acetate from *Dioscorea bulbifera* has been proven to cure the antibiotic-resistant R-plasmids of isolates of *E. faecalis*, *E. coli*, *Shigella sonnei*, and *P. aeruginosa*, with an average curing efficiency of 34% [104].

In summary, the ability of phytochemicals to combat ARB and their mechanisms of action have been demonstrated at various experimental levels, from in vitro to clinical tests. The research, discovery, and development of new formulations based on safe, natural materials such as phytocompounds will continue to be a highly attractive research topic.

## 6. Syntheses of Chitosan–Phytochemical Composites

The utilization of synthetic materials based on chitosan has been increasingly researched and applied, particularly in the food industry and biomedical fields [105]. Being a natural polysaccharide, chitosan possesses both hydroxyl and amino functional groups, facilitating its facile conjugation with bioactive compounds and thereby contributing to the diversity in structure and function of synthesized chitosan composites [106].

In fact, other polysaccharides, such as alginate, can act as a carrier of phytochemicals in discovering novel bioactive composites. Alginate contains carboxyl (-COOH) and hydroxyl (-OH) groups, supporting ionic and hydrogen bonding interactions. However, the mechanical strength of the alginate–phytocompound complex is basically low and requires cross-linking agents such as calcium ions for enhanced strength. Unlike alginate and other polysaccharides, chitosan contains reactive amino (-NH_2_) and hydroxyl (-OH) groups, while phytochemicals possess hydroxyl (-OH) and carbonyl (-C=O) groups. These functional groups enable both covalent and non-covalent interactions, contributing to the formation of stable and bioactive composites. Specifically, the amino (-NH_2_) group of chitosan reacts with the carbonyl (-C=O) group of phytochemicals through a condensation reaction, forming a -C=N- imine linkage, which significantly enhances the stability and antimicrobial activity of the composite. Additionally, hydroxyl (-OH) groups from both chitosan and phytochemicals facilitate hydrogen bonding, providing further structural stability. Under specific conditions, hydroxyl groups may also participate in esterification reactions, further strengthening the composite structure. These interactions collectively enhance the mechanical and chemical stability of the chitosan–phytochemical composite while improving its bioactivity, particularly its antimicrobial properties, as demonstrated in Table 4.

**Figure 4:**
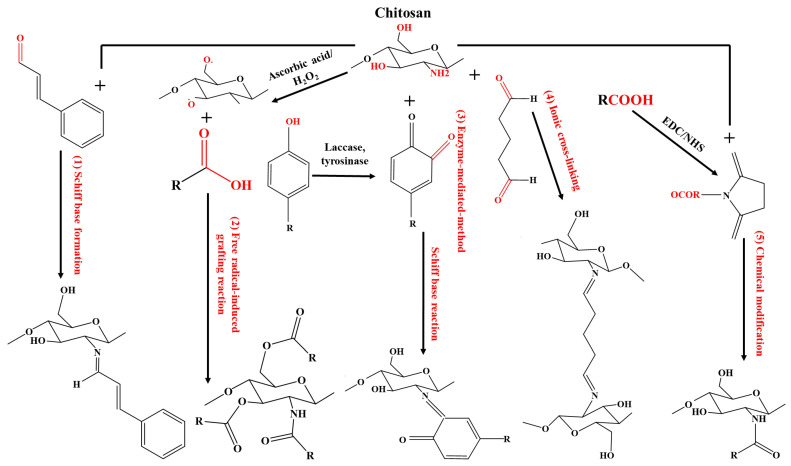
Schematic representation of the grafting reaction between chitosan and phytochemicals. Functional groups and corresponding synthetic strategies are marked in red.

Several common methods for the synthesis of chitosan–antimicrobial conjugates have been reported, including (1) Schiff base formation [107], (2) free radical-induced conjugation [106], (3) enzyme-assisted coupling reactions [23,108], (4) ionic cross-linking [62], and (5) chemical modification [106], as depicted in Figure 4.

Most of these methods are selectively employed with phytochemicals, among which phenolic compounds have been widely studied. Table 4 summarizes some common synthesis methods for CPCs along with their corresponding phytochemicals.

**Table 4:** Common synthesis methods of chitosan–phytochemical composites.

Synthetic Techniques	Catalysts	Phytochemicals	References
Schiff base formation	4-Dimethylaminobenzaldehyde	Medicinal plants	[107]
Free radical-induced grafting reaction	Ascorbic acid/hydrogen peroxide redox pair	Gallic acid, caffeic acid, ferulic acid, catechin, epigallocatechin gallate, phloroglucinol	[22,109,110,111,112,113,114,115]
Enzyme-mediated-method	Laccase, tyrosinase, chloroperoxidase	Gallic acid, caffeic acid, ferulic acid, tannic acid, catechin, quercetin, flavonols	[22,109,110,111,112,113,114,115]
Ionic cross-linking	Acetic acid	Cinnamaldehyde, glutaraldehyde	[62]
Chemical modification	EDC; EDC/NHS	Gallic acid, caffeic acid, ferulic acid, salicylic acid, protocatechuic acid, hydroxycinnamic acid, hydroxybenzoic acid	[112,115,116,117,118,119,120,121]
	CAN	Eugenol	[122]

EDC: 1-Ethyl-3-(3-dimethylaminopropyl) carbodiimide; EDC/NHS: 1-Ethyl-3-(3-dimethylaminopropyl) carbodiimide/*N*-hydroxysuccinimide; CAN: Ceric ammonium nitrate.

In this study, we propose a new strategy for synthesizing CPCs to optimize the compatibility, stability, and activity of the final product. Unlike other reported methods, this report focuses on the careful selection of phytochemical candidates with strong and stable properties, particularly in terms of the structural integrity and composition of functional groups in the synthesis reactions. Spectroscopic techniques such as nuclear magnetic resonance (NMR), infrared (IR), and mass spectroscopy (MS) serve as tools utilized in qualitative methods and quality control of chitosan conjugates under various conditions such as temperature, pH, UV, etc. Ultimately, an essential step involves the evaluation of the activity of the synthesized composites and their practical applications. A typical synthesis strategy for chitosan–phytochemical conjugates includes the following steps.

- Selection of phytochemical candidates: The careful selection of phytochemicals is imperative and contingent upon the specific intended application. Various plant extracts and bioactive compounds, renowned for their distinct properties such as antioxidant, antimicrobial, and anti-inflammatory effects, should be meticulously chosen through both in vitro and in vivo assays.
- Preparation of chitosan solution: Chitosan is typically dissolved in an acidic solution. The selection of solvents profoundly influences the solubility and characteristics of the resultant CPCs.
- Incorporation of phytochemicals: Phytochemicals are then introduced into the chitosan solution, ensuring homogeneity. This step may involve stirring, sonication, or other methods to facilitate thorough blending and ultimately optimize the amalgamation.
- Composite formation: The composite is formed by allowing the solvent to evaporate, leading to the coalescence of chitosan and phytochemicals. Techniques such as casting, freeze-drying, or electrospinning are employed based on the desired structural configuration of the composite.
- Characterization: The synthesized composite is characterized using various analytical techniques, including spectroscopy, microscopy, and mechanical testing. This step helps assess the structural integrity, chemical composition, and functional properties of the composite.
- Evaluation of stability and biological activities: The stability of the synthesized composites is evaluated under diverse environmental conditions, while the biological activity of the product is scrutinized both in laboratory settings and actual applications.

## 7. Inhibition of Antibiotic-Resistant Bacteria by Chitosan–Phytochemical Composites

In this section, several examples of using CPCs to inhibit ARB growth are reported (Table 5).

Table 5 highlights the potential of CPCs in combating bacteria that are widely known for their antibiotic resistance. The data demonstrate that CPCs exhibit greater antimicrobial activity than UC. This can be explained by the interactions or synergistic effects of chitosan and phytochemicals, which may enhance the inhibition of bacteria through an increase in the targets of action [81]. The findings may provide an effective approach to addressing the challenges posed by ARB. Consequently, the subsequent section will delve into their applications in ensuring food safety. The differences in the modes of antibacterial activity have been demonstrated. It was reported that caffeic acid–chitosan conjugates could quench free electrons from the electron transport chain, altering the electric potential of the bacterial membrane. Additionally, these conjugates can reduce or completely inhibit bacterial growth by interfering with the proton efflux pump as a result of their interaction with the dehydrogenase enzyme [123]. Hydroxycinnamic acid-grafted chitosan can disrupt cell membranes and cause cytoplasmic leakage [124].

These studies have revealed that CPCs are promising candidates for controlling ARB. However, in practical applications, CPCs have faced challenges such as sensitivity to environmental factors (e.g., humidity and pH), which can impact their stability and mechanical durability [125]. Furthermore, despite being considered safe, in vivo tests, followed by clinical trials, as well as long-term studies, are required to ensure the safety of these candidates for widespread use. On the other hand, the strong flavor of some phytochemicals can affect the sensory quality of food, requiring careful formulation [97]. Continuous research on specific food types (e.g., fish, meat, and postharvest products) is also essential to confirm the potential of CPCs.

## 8. Application of CPCs as Surface Coatings for Food Preservation

### 8.1. Preparation Techniques for Chitosan–Phytochemical Composite Coatings/Films

#### 8.1.1. Chitosan–Phytochemical Composite-Based Coatings

**Figure 5:**
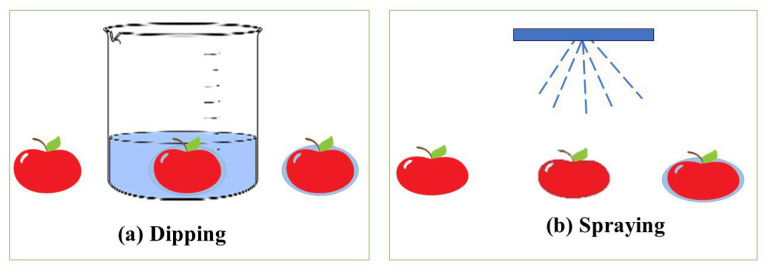
Dipping (**a**) and spraying (**b**) techniques for applying coatings to food.

Coatings refer to the application of a layer or covering on a surface of food using different techniques such as spraying, dipping, or spreading (Figure 5). CPC coating serves as a valuable technique for inhibiting microbial growth, extending shelf life, and preserving overall product quality [126].

The coating procedure involves several sequential steps [127,128,129,130]:

1. Preparation of raw materials by blending appropriate proportions of chitosan and fillers;
2. Creation of coating samples using various methods such as irradiation, heating, mixing, and steam flash pasteurization;
3. Sanitization of food samples using sodium hypochlorite;
4. Application of chitosan-based composite solutions to food using a sterile spreader;
5. Drying under specific conditions;
6. Packaging and storage in suitable environments.

Antimicrobial components have the potential to migrate from the films into the food, thereby extending the shelf life of the food. These techniques are employed in the coating of fruits and vegetables to enhance their longevity [128].

#### 8.1.2. Chitosan–Phytochemical Composite-Based Film

**Figure 6:**
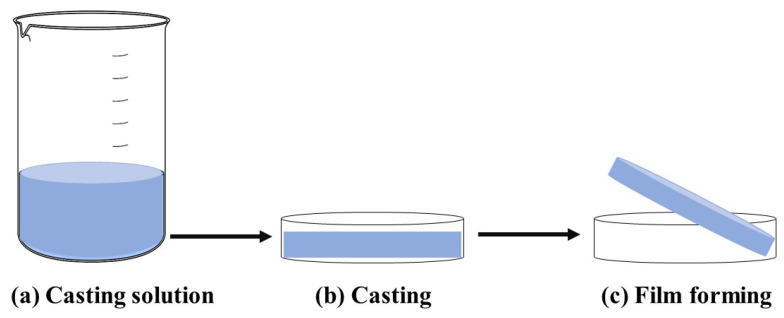
Film forming made from chitosan–phytochemical composites.

Film forming refers to the process of creating a film or coating by dissolving chitosan in a solution and then casting or spreading the solution to form a solid film as the solvent evaporates [129,131]. The procedure is cost-effective and straightforward, leading to the formation of the polymer structure through intermolecular electrostatic and hydrogen bonding [130]. The typical approach for producing the film through the solution casting method is shown in Figure 6.

The fabrication process comprises multiple steps. (1) Initially, to produce the casting solution, chitosan is dissolved in an acidic solution with a pH below 6.0. This solution subsequently undergoes blending, mixing, or crosslinking with other biopolymers, fillers, and functional materials in varying proportions. The resulting mixture is then stirred to achieve a homogeneous viscous solution, followed by processes such as filtration, sonication, or centrifugation to eliminate air bubbles and insoluble particles. (2) Once prepared, the solution is cast or poured onto a surface for drying in the casting phase. (3) Finally, after complete drying, the resulting film is peeled off [132,133]. As a consequence, the produced edible film is employed to wrap the surface of a food product and has positive effects on the preservation of postharvest products [134,135].

#### 8.1.3. Chitosan–Phytochemical-Based Layer-by-Layer Edible Coatings

**Figure 7:**
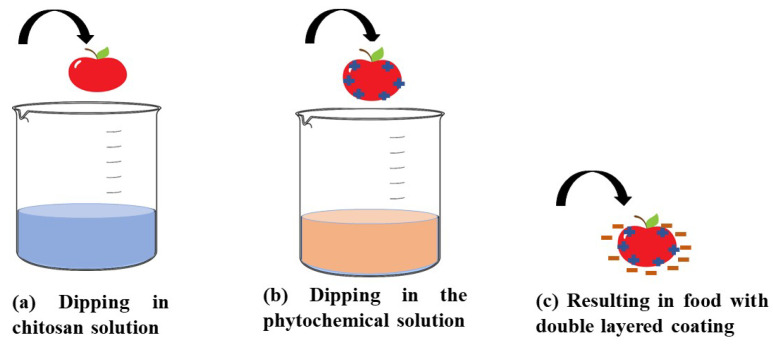
Steps for the layer-by-layer method of coating food.

The layer-by-layer method for coating food is an advanced technique that entails the sequential deposition of alternating layers of materials onto food products, resulting in a multilayered coating, as illustrated in Figure 7. This approach offers precise control over the composition, thickness, and functionality of the coating, making it particularly advantageous for various food applications [133].

This technique for creating multi-layered films eliminates the need for intricate equipment. Within the layer-by-layer assembly paradigm, surface modification primarily depends on the mutual attraction and deposition of alternating polyelectrolytes. Layer-by-layer deposition offers the opportunity to formulate active packaging films and coatings by incorporating active agents. It has been suggested that the layer-by-layer method, when used synergistically with other techniques, effectively preserves food quality and extends shelf life [136,137,138].

### 8.2. Application of Composite Coatings/Films in the Food Industry

#### 8.2.1. Application in the Preservation of Fish

Chitosan has demonstrated its effectiveness in extending the shelf life of fish and fishery products by inhibiting the growth of spoilage bacteria and preserving product quality [139]. In another study, a coating composed of chitosan and curcumin nanoparticles significantly reduced bacteria counts and prolonged the shelf-life of fish filletsup to 15 days [140]. Ehsani et al. explored the effects of chitosan films combined with sage essential oil on the deterioration of fish burgers made from common carp flesh. The coatings effectively inhibited or slowed the growth of harmful and spoilage-causing bacteria, with sage essential oil preventing the production of off-flavors [141].

#### 8.2.2. Application in the Preservation of Meat

When bacteria infiltrate the inner layers of meat, it is challenging to eliminate them through cooking. Deterioration caused by microorganisms is the primary factor affecting the quality and shelf life of meat. Packaging materials made from antimicrobial substances are now prioritized over artificial food preservatives. Hence, several studies have been undertaken to develop films based on CPCs and to employ them in the processing of meat products, including red meat, poultry, and pork [142]. Green tea extract has been incorporated into a chitosan-based film as an active component to prolong the shelf life of pork sausages. The results indicated that pork sausages enveloped in a chitosan film containing green tea extract demonstrated fewer alterations in color, texture, 2-thiobarbituric acid value, sensory attributes, and microbial growth compared to the control group. Ultimately, the study suggested that the addition of green tea extract to the chitosan film could enhance its antioxidant and antibacterial properties, thereby preserving the quality and extending the shelf life of pork sausages [143]. In another study, Gaba et al. investigated the efficacy of chitosan films combined with essential oils of oregano and thyme in mitigating meat deterioration and inhibiting harmful microorganisms. The produced films successfully suppressed the proliferation of psychrophilic bacteria [144].

#### 8.2.3. Application in the Preservation of Postharvest Products

The utilization of natural preservatives in the preservation of postharvest products has become increasingly crucial to enhance food safety, extend shelf life, and minimize food waste. The application of edible coatings and films based on CPCs has gained prominence in postharvest preservation. Edible coatings contribute to preserving fruits and vegetables while offering a sustainable alternative to traditional packaging materials. The use of biopolymer-based edible films for wrapping or coating presents an affordable, straightforward, and effective solution to prevent moisture loss and reduce degradation and respiration rates. Additionally, preserving minimally fresh-cut products presents a significant challenge due to their rapid deterioration. This is primarily driven by increased respiration rates, elevated ethylene production, and accelerated consumption of sugars, lipids, and organic acids during the ripening process. These changes can result in texture degradation, moisture depletion, and undesirable alterations in flavor and color associated with senescence. To address these spoilage issues, applying edible films or coatings can be effective. These films form a semi-permeable barrier to gases and water vapor, thereby maintaining food quality and extending shelf life while enhancing the appearance, flavor, color, and nutritional value of the products [145]. Studies have shown that incorporating chitosan-based composites with bioactive compounds is advantageous in extending the shelf life and maintaining the quality of postharvest products [146]. For instance, a chitosan composite film containing apple peel polyphenol increased the shelf life of strawberries [147]. A chitosan–salicylic acid composite maintained the quality of fresh-cut cucumbers and improved their shelf life [148]. Similarly, a chitosan–ascorbic acid composite extended the storage period of fresh-cut apples [149]. In the case of mushrooms, a chitosan–thyme oil composite successfully prolonged their shelf life [150]. For fresh-cut potatoes, chitosan coatings with cinnamon essential oil have been developed to enhance quality and microbiological safety [151]. Similarly, Yang et al. employed a chitosan coating comprising blueberry leaf extract to enhance the preservation of fresh blueberries [152]. These examples highlight the versatility and effectiveness of chitosan-based composites in fresh produce preservation.

CPC coatings are considered to contribute to the preservation and protection of food from production to consumption [105,116,140,146]. Particularly, CPC coatings inhibit bacterial growth on food surfaces, creating a protective barrier that helps to prevent or significantly reduce the risk of foodborne illnesses. By actively suppressing the proliferation of harmful bacteria, CPC coatings contribute to enhanced food safety and extend the shelf life of food [130,153]. Additionally, these coatings help maintain the freshness and nutritional quality of the food throughout transportation and storage [130,153]. CPC coatings can also reduce the risk of cross-contamination during handling, packaging, and transportation, protecting food from harmful bacteria [22,141,142]. In another aspect, despite good practices (e.g., washing, cooking, or processing before consumption) can reduce the risk of bacterial contamination and food poisoning, these practices require stringent and rigorous standards, such as the hygiene of the person handling the food, cooking utensils, and water used during cooking, among other factors [154]. Furthermore, considering the widespread presence of microorganisms and multiple pathways of food contamination, it is challenging to assert that food processed in such a manner is completely free of bacteria [154]. While improper washing, cooking, or processing can adversely affect food safety, the pre-storage preservation of food before it reaches the consumer is a crucial step. Consequently, the application of CPC coatings shows significant potential in preventing the accumulation of bacteria and their toxins, as well as reducing the risk of contamination by pathogenic bacteria, particularly ARB. On the other hand, it is noteworthy that combining multiple control processes and safety measures simultaneously will yield optimal results in protecting consumer health [154]. Therefore, CPC coatings can act as an additional measure to enhance food safety alongside good practices. For example, if bacteria are inhibited or minimized by the coating, cooking will be more effective in eliminating remaining bacteria in food [154]. Additionally, for uncooked products such as raw vegetables, fresh fruits, and sushi, the use of protective coatings is crucial as they are not cooked before consumption [155]. Similarly, processed or convenient foods require protective coatings to maintain freshness and safety before consumption [156]. Studies suggest that CPC coatings can be a natural alternative to synthetic chemical preservatives, helping reduce the negative impact on consumer health [157].

To effectively apply CPCs in food preservation and ensure food safety, it is essential to select the appropriate CPC formulation for each type of food. Achieving this requires thorough data collection on bacterial contamination risks, including information on ARB found in specific food types, such as meat, vegetables, fish, and dairy, as detailed in Table 1. Subsequently, bacterial contaminants can be detected using traditional culture-based methods and rapid molecular techniques [158]. Following detection, model simulations, such as multi-factorial models, ComBase models, bi-dimensional and tri-dimensional models, and Baranyi or Gompertz models, can be employed to simulate bacterial growth under various conditions, including initial contamination levels, storage conditions, and handling methods [159,160]. Based on the simulation results, CPC research can then be conducted to identify the most effective composite against the predicted bacteria. For example, if a high risk of *E. coli* contamination is identified, CPCs containing quercetin [23] or gallic acid [21], known to inhibit *E. coli*, might be selected. CPC optimization involves adjusting the concentration and application method to maximize effectiveness against the identified bacteria. Experimental testing under real-world conditions follows, where the effectiveness of the CPC is tested against the predicted bacterial strains, and the simulation model is refined based on the experimental results. Continuous evaluation and improvement are critical, involving data analysis to compare experimental results with simulations and refining the model and methods to optimize CPC effectiveness in food preservation. Ultimately, using simulation and prediction methods helps identify the bacterial strains most likely to contaminate food, enabling the selection of an appropriate CPC for preservation, thereby enhancing the effectiveness of food preservation and safety.

#### 8.2.4. Application in Intelligent Packing (IP) Technology

Recently, smart agriculture, via the application of advanced technologies, has been considered the future of global agricultural production. In addition to increasing productivity and the quality of agricultural products, the preservation of these products using IP technology has been receiving rising interest worldwide [161]. For example, IP films have been developed based on the fortification of natural polymers (e.g., starch, cellulose, chitosan, pectin, etc.) with pH-sensing materials [162,163]. Thus, IP films feasibly monitor the quality and freshness of packed food in real-time through the visible color adjustment due to pH change via the spoilage process, especially in proteinaceous foods [164]. This packing technology helps food supply chain managers control products during storage, transportation, and distribution. Additionally, consumers can easily make better decisions regarding food freshness and quality. In the past, chemical substances such as cresol red, bromophenol blue, bromocresol green, etc., were used as pH colorimetric indicators for producing IP films [165]. However, safety concerns are a major limitation in applying these synthetic dyes [165]. Therefore, an alternative source of pH indicator for IP films can be mentioned as anthocyanins derived from plants. These natural pigments are stable under acidic conditions and unstable at higher pH levels [166]. In addition to anthocyanins, natural products such as essential oils, phenolics, fatty acids, terpenes, flavonoids, and steroid extracts from plants can be directly added to the film-forming matrix, which can improve pH sensing and water vapor resistance [167]. Moreover, these plant-based materials can satisfy safety requirements, as well as contribute to the antioxidant, antibacterial, and antifungal activities, which may enhance the economic properties of the film [167]. Accordingly, these substances are promising candidates for the development of smart packaging technology.

In terms of CPCs, while some investigations have explored the potential of CPCs for active packaging, their direct integration into IP systems remains under development. In the context of the rapid advancements in material science and artificial intelligence (AI), IP technology is expected to evolve beyond conceptual frameworks and soon find practical applications. Potential approaches include developing property models for various food types, establishing comprehensive datasets of possible pathogens, and using these to simulate CPC structures tailored to the specific surface characteristics of each food. Such approaches aim to optimize binding affinity and enhance the practical functionality of CPCs in actual IP applications.

## 9. Current Status, Existing Limitations, and Future Perspectives

The emergence of ARB presents a significant challenge to both public health and food safety. Consequently, there has been a notable interest in exploring alternative antimicrobial agents. This article provides a comprehensive review of chitosan, a natural biopolymer derived from chitin, which has demonstrated antimicrobial properties, rendering it an appealing candidate for addressing ARB. When combined with phytochemicals sourced from plants, this composite presents a synergistic effect, enhancing antimicrobial activity. Current research has yielded promising findings, indicating that CPCs possess antimicrobial properties capable of inhibiting the growth of antibiotic-resistant bacterial strains commonly encountered in foodborne pathogens. Furthermore, studies have illustrated the effective application of these composites across diverse food matrices, encompassing meats, fishes, fruits, and vegetables, to mitigate microbial contamination and extend shelf life. The integration of natural compounds like chitosan and phytochemicals derived from crop residues and agricultural by-products aligns with the growing need for sustainable and eco-friendly substitutes to traditional antibiotics in food production systems. This direction holds promise and is deemed appropriate.

Future research should prioritize overcoming existing limitations and optimizing the efficacy of these composites. To date, research has primarily focused on chitosan composites with phenolic compounds, while studies involving chitosan composites with other phytochemical groups, such as terpenoids (including carotenoids and triterpenes) and alkaloids, remain limited. Furthermore, a deeper understanding of the mechanisms underlying the antimicrobial action of these composites is necessary to enhance their effectiveness against antibiotic-resistant bacteria (ARB). Standardization of methodologies and thorough characterization of the composites are crucial to ensure the reproducibility and comparability of research findings.

For future considerations, CPC coatings present significant potential for diverse applications across various fields, particularly in the food industry, due to their strong antibacterial and antioxidant properties [168,169]. These coatings are biocompatible, safe for human use, environmentally friendly, and biodegradable [105]. Their flexibility allows for a wide range of applications, from antimicrobial food packaging [170] to preservation coatings [171]. In the field of nanotechnology, chitosan–phytochemical nanoparticles, with their increased surface area compared to larger particles, can interact more effectively with bacteria, cells, and other molecules, thereby enhancing their antibacterial efficacy, including against ARB [81]. These nanoparticles can be employed to extend the shelf life of food [130], inhibit the growth of harmful microorganisms [81], and protect consumer health by delivering antioxidants and beneficial plant compounds [172,173]. Addressing the regulatory aspects related to the safety and approval of these novel antimicrobial agents for food use is also essential. Collaborative efforts among researchers, food manufacturers, and regulatory agencies will be vital for advancing this field and facilitating the adoption of CPCs as sustainable alternatives to conventional antibiotics in food production. Additionally, exploring innovative delivery systems and applications, such as incorporating these composites into food packaging materials, could provide the dual benefits of food preservation and antimicrobial protection.

In agricultural production, crop residues and by-products pose significant environmental, economic, and social challenges [161]. The effective utilization of these resources has recently become a global priority, contributing to the sustainable development of agriculture [160]. Notably, crop residues and by-products have been shown to contain numerous bioactive compounds, such as anthocyanins, phenolics, flavonoids, fatty acids, terpenes, and steroids, which can be valuable for producing IP films [161]. Recent advancements in extraction and isolation methods have improved the ability to obtain these compounds from plant sources through simple, convenient, and cost-effective procedures [161,174,175,176]. Therefore, the efficient use of crop residues and by-products could lead to the availability of IP materials, potentially reducing global pollution caused by the overuse of traditional packaging microplastics.

Overall, whether in laboratory research or industrial-scale applications, the stability and performance of CPCs are crucial for their effectiveness in applications such as food preservation. Phytochemicals, being sensitive to light, UV radiation, and humidity, benefit from incorporation into the chitosan matrix, which provides a protective barrier against environmental stressors. In a complex, chitosan can shield phytochemicals from light and UV exposure while reducing their interaction with oxygen and moisture. Further stabilization can be achieved through encapsulation techniques, pH adjustments, and the use of cross-linking agents, which enhance the interaction between chitosan and phytochemicals. Forming chemical bonds, such as Schiff base (-C=N-) linkages or ester bonds, significantly improves the stability and retention of bioactive properties. Evaluating the composite involves assessing loading efficiency, release profiles, resistance to environmental factors, and functional bioactivities such as antimicrobial or antioxidant effects. These strategies collectively ensure that chitosan–phytochemical composites maintain their stability and functional performance, making them promising materials for food preservation.

In practical production and application, addressing the surface characteristics of different foods is crucial to enhancing the applicability and effectiveness of CPCs in food preservation. Food surfaces exhibit varying hydrophilic or hydrophobic properties depending on their composition. For example, vegetable products often have waxy or cuticle layers that contribute to hydrophobicity, while fruit surfaces may vary in texture and chemical composition, affecting coating adherence. In contrast, meat surfaces are primarily hydrophilic, consisting of proteins and moisture, which necessitate tailored coating formulations for stable adhesion. CPC formulations must be designed to align with the specific surface properties of each type of food, a task that can be supported by advanced simulation technologies integrated with AI.

Additionally, the distribution and attachment of pathogenic bacteria differ significantly between food surfaces and interiors. External contamination on surfaces can be more effectively managed with coatings, whereas internal microbial contamination requires preventive measures during harvesting, processing, and packaging. Furthermore, advancements in genetic technologies may enable the development of new plant and animal varieties with superior antimicrobial traits compared to traditional breeds, providing another layer of defense against microbial contamination.

## Figures and Tables

**Table 1:** Antibiotic-resistant bacteria in food and their resistance to antibiotics.

Bacterial Strains	Food Sources	Antibiotic Resistance	References
*Staphylococcus aureus*	Poultry meat	Methicillin	[39]
*Escherichia coli*	Meat	Tetracycline, trimethoprim/sulfamethoxazole, cefazolin	[40]
*Bacillus subtilis*	Raw milk	Cefotaxime, ampicillin, rifampicin, and norfloxacin	[41]
*Enterococcus faecalis*	Cheese	Rifampicin, vancomycin, teicoplanin, erythromycin, minocycline, nitrofurantoin	[42]
*Listeria monocytogenes*	Ready-to-eat foods	Amoxicillin, penicillin, ertapenem, erythromycin, sulfamethoxazole	[43]
*Klebsiella pneumoniae*	Raw bean sprouts	Ceftriaxone	[44]
*Pseudomonas aeruginosa*	Chicken, pork	β-Lactams, cephalosporins, and carbapenem	[45]
*Salmonella enterica* serovar Typhimurium	Chicken	Ampicillin, streptomycin, sulfonamides, and tetracycline	[46]
*Shigella flexneri*	Vegetable salad, ground meat, and raw cow’s milk	Sulfamethoxazole/trimethoprim, amoxicillin, streptomycin, tetracycline, ampicillin	[47]

**Table 2:** Chitosan properties and applications.

Properties	Application	References
Film-forming	Postharvest fruit preservation as coating agent Wound healing Tissue engineering (bone) Drug delivery	[51,54,55,56]
Biodegradability	Drug delivery Food packaging	[57,58]
Antimicrobial activity	Food preservation Infection control	[18,19]
Hypolipidemic activity	Treatment of cardiovascular disease	[59]
Immunoadjuvant activity	Immunomodulatory agent	[60,61]

**Table 3:** Phytochemicals and antibacterial activity.

Phytochemical Groups	Antimicrobial Agents	Targeting Bacteria	References
Terpenoids	Carotenoids (β-carotene, torulene, and torularhodin)	*E. coli*, *P. aeruginosa*	[82]
	Diterpenoids (momilactones)	*P. ovalis*, *B. cereus*, *B. pumilus*, *E. coli*	[17]
	Triterpenes (phytosterols)	*E. coli*, *S. typhi*	[83]
Phenolics	Caffeic acid	*S. aureus*	[84]
	Salicylic acid	*E. coli*, *S. aureus*	[85]
	Lauric acid	*Bacteroides*, *Clostridium*	[86]
	Ellagic acid	*Helicobacter pylori*	[87]
	Ferulic acid	*S. aureus*	[88]
	Gallic acid	*E. coli*	[89]
	Curcumin	*E. faecalis*, *K. pneumoniae*, *P. aeruginosa*, *B. subtilis*, *S. epidermidis*, *B. cereus*, *S. aureus*, *E. coli*, *S. enterica*	[90]
	Eugenol	*S. aureus*	[91]
	Cinnamaldehyde	*S. aureus*	[92]
	Flavonoids (quercetin)	*S. aureus*	[93]
Alkaloids	Caffeine	*P. aeruginosa*	[94]

**Table 5:** MIC of chitosan–phytochemical composites against ARB.

Strain	MIC (μg/mL)			References
	UC	CPCs		
*S. aureus*	128	Chitosan–gallic acid	32	[21]
*B. subtilis*	64		16	
*B. cereus*	128		32	
*E. faecalis*	64		16	
*L. monocytogenes*	128		16	
*E. coli*	1024		256	
*K. pneumoniae*	512		128	
*P. aeruginosa*	512		256	
*S. enterica serovar* Typhimurium	512		128	
*S. flexneri*	1024		256	
*P. acnes*	16	Chitosan–caffeic acid	256	[22]
*S. epidermidis*	64		8	
*S. aureus*	32		64	
*P. aeruginosa*	32		16	
*P. acnes*	512	Chitosan–ferulic acid	256	[22]
*P. acnes*	512	Chitosan–sinapic acid	256	[22]
*P. aeruginosa*	18 mm *	Medicinal plants tagged with chitosan	20 mm *	[107]
*S. aureus*	19 mm *		22 mm *	
*E. coli*, *P. aeruginosa*, *S. aureus*	Three orders of magnitude	Quercetin-modified chitosan	Seven orders of magnitude (antimicrobial activity twice as high as that of UC)	[23]

UC: Unmodified chitosan; * Zone of inhibition/mm.
